# Schedule-dependent activity of 5-fluorouracil and irinotecan combination in the treatment of human colorectal cancer: *in vitro* evidence and a phase I dose-escalating clinical trial

**DOI:** 10.1038/sj.bjc.6603496

**Published:** 2006-12-12

**Authors:** C Barone, M Landriscina, M Quirino, M Basso, C Pozzo, G Schinzari, G Di Leonardo, E D'Argento, N Trigila, A Cassano

**Affiliations:** 1 Clinical Oncology Unit, Department of Internal Medicine, Catholic University, Rome, Italy; 2 Clinical Oncology Unit, Department of Medical Sciences, University of Foggia, Foggia, Italy

**Keywords:** 5-fluorouracil, irinotecan, SN-38, colon carcinoma cells, phase I trial

## Abstract

Several schedules of 5-fluorouracil (FU) and irinotecan (IRI) have been shown to improve overall survival in advanced colorectal cancer (CRC). Preclinical evidence suggests that the sequential administration of IRI and FU produces synergistic activity, although their clinical use has not been fully optimised. We investigated the interaction between short-term exposure to SN-38, the active metabolite of IRI, and prolonged exposure to FU in human CRC HT-29 cells and observed that the synergism of action between the two agents can be increased by extending the time of cell exposure to FU and reducing the interval between administration of the two agents. Based on these findings, we performed a phase I trial in 25 advanced CRC patients using a modified IRI/FU regimen as first-line therapy and evaluated three dose levels of IRI (150–300 mg/m^2^) and two of continuous infusion of FU (800–1000 mg/m^2^) in a 3-weekly schedule. The most severe grade III–IV toxicities were neutropoenia in four cycles and diarrhoea in three. One patient achieved complete response (4%), 12 a partial response (48%), the overall response rate was 52% (±20, 95% CI); seven of 25 patients had stable disease (28%), the overall disease control was 80% (±16, 95% CI). This modified IRI/FU schedule is feasible and exhibits potentially interesting clinical activity.

Combination therapy with FU, IRI and oxaliplatin (l-OHP) administered in two- or three-drug regimens is the mainstay of treatment for advanced CRC (Venook, 2005; O'Neil and Goldberg, 2005). Indeed, regimens combining FU with IRI or l-OHP are equally effective in terms of response rate and overall survival (Tournigand *et al*, 2004) and represent the standard first-line treatment in advanced CRC (O'Neil and Goldberg, 2005). Although three combinations of IRI and FU (IFL, FOLFIRI and AIO+IRI) have been evaluated in phase III studies (Saltz *et al*, 2000; Douillard *et al*, 2000; Köhne *et al*, 2005), several other schedules have been proposed (Venook, 2005; Atalay *et al*, 2003). However, no randomised clinical trials have compared these different FU/IRI schedules, so that we do not know whether one regimen is better than others.

The most effective regimens combining FU and IRI were designed on the basis of preclinical evidence suggesting that the antiproliferative activity of the two agents is schedule-dependent (Guichard *et al*, 1997; Guichard *et al*, 1998; Mullany *et al*, 1998). Indeed, the administration of IRI before FU produced additive or synergistic effects in all colon carcinoma cell lines tested (Guichard *et al*, 1998; Mans *et al*, 1999), whereas both the exposure of cells to FU before IRI and the simultaneous administration of both drugs produced antagonistic or only additive activity, depending on the colon tumour cell model (Mans *et al*, 1999). Similar findings were also reported *in vivo*, in athymic mice xenografts of colon carcinoma cells (Guichard *et al*, 1997).

Several combination regimens with FU and IRI have been evaluated as first-line therapy for advanced CRC, achieving a response rate of 30–50% and an overall survival of 14–20 months (Venook, 2005; O'Neil and Goldberg, 2005). Interestingly, the most commonly used schedules – that is the FOLFIRI and IFL regimens – consist in the sequential administration of IRI followed by FU bolus and/or continuous infusion (c.i.). However, considering that the half-life of IRI is about 10 h (Robert and Rivory, 1998), both regimens also combine the two drugs simultaneously in a weekly (IFL regimen) or bi-weekly (FOLFIRI regimen) schedule (Douillard *et al*, 2000; Saltz *et al*, 2000). While there is no clinical evidence to prove that the drug interactions observed in preclinical models also occur in humans and may affect the effectiveness of FU- and IRI-based chemotherapy, the results obtained *in vitro* clearly indicate a schedule-dependency of the interaction between the two agents and suggest the possibility of improving the efficacy of the combination. We therefore looked more closely at the dependency of the synergism between FU and SN-38, the active metabolite of IRI, on the extent of cell exposure to FU and the interval between the two drugs in human colon carcinoma HT-29 cells and translated the findings obtained *in vitro* into clinical experience, evaluating a modified IRI/FU regimen in a phase I trial.

## PATIENTS AND METHODS

Cell Cultures and Chemicals. HT-29 human colon carcinoma cells were cultured in DMEM containing 10% foetal bovine serum, glutamine and Penicillin/Streptomycin (Sigma-Aldrich, Italy) (Khatib *et al*, 2001). SN-38 was kindly provided by Aventis Inc., Paris, France. Stock solution of SN-38 was prepared in DMSO at 10 mM. FU was purchased from Sigma-Aldrich and diluted in phosphate buffer saline. Either drugs or the same DMSO volume were added to the cultures at the concentrations specified in the Results; incubation was carried out continuously and fresh drug-containing medium was changed at 24-h intervals.

The cell-cycle phase distribution and the rate of necrosis and apoptosis were evaluated as previously reported (Landriscina *et al*, 2000; Sciamanna *et al*, 2005).

In order to select HT-29 colon carcinoma cells resistant to FU (HT29 FUR) or to SN-38 (HT29 SN-38R), cells were continuously incubated in the presence of increasing concentrations of FU and SN-38 starting from 0.1 nM for both drugs (Lesuffleur *et al*, 1991). Cells were finally stabilised in the presence of 9 *μ*M FU and 100 nM SN-38. The resistance to each drug was assessed by MTT dye assay (see below) and by measuring apoptosis in the presence of increasing concentrations of FU or SN-38.

### Immunoblot analysis and cytotoxicity assay

Immunoblot analysis was performed as previously reported (Landriscina *et al*, 2000). Specific bands were revealed using a mouse monoclonal anti-thymidylate synthase (TS) antibody (Histoline Laboratories, Italy).

Growth inhibition by cytotoxic agents was measured using the MTT (Sigma-Aldrich, Italy) dye assay as previously described (Zeghari-Squalli *et al*, 1999). Briefly, 10^4^ cells were seeded into 24-well plates and incubated, 24 h later, in the presence of increasing concentrations of FU (10^−4^−100 *μ*M) or SN-38 (10^−6^−10 *μ*M) as specified in the Results. Three independent cytotoxicity assays were used to calculate EC_30_ SN-38 and EC_30_ FU. Combination assays were performed using EC_30_ SN-38 (0.11 *μ*M) or FU (5.2 *μ*M) with increasing concentrations of FU or SN-38, respectively. SN-38 was always administered for 6 h, whereas FU was administered for 24–96 h, as specified in the Results. In the sequential schedule, the second drug was administered immediately after the first drug or, in some experiments, after an incubation of cells in a drug-free medium for 24–96 h. After the removal of both drugs, cells were incubated in a drug-free medium for 72 h and 50 *μ*l of a 125 *μ*M MTT solution were then added to each well. The plates were incubated for additional 3 h at 37°C to allow MTT metabolism into formazan crystals. The formazan crystals were finally solubilised by adding 200 *μ*l of 0.04 N HCl in isopropanol to each microplate well. Adsorbance at 540 nm was measured using a Bio-Tek microplate reader (model EL-340; BioMetallics, Priceston, NJ, USA). Wells containing only DMEM, 10% FBS and MTT were used as controls. Each experiment was performed at least three times, using four replicates for each drug concentration.

### Analysis of combination effects

Combination analysis was performed using the method described by Chou *et al.* (Chou and Talalay, 1984; Chou *et al*, 1994). The influence of the two drugs on the combination was evaluated by comparing the sequential assay with assays involving the two drugs simultaneously or alone. The combination effect was evaluated from isoeffect analysis CIs, calculated as follows: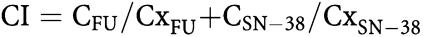 where Cx_FU_ and Cx_SN-38_ are respectively the concentrations of FU and SN-38 alone needed to achieve a given effect (*x*%) and C_FU_ and C_SN-38_ are the concentrations of the two drugs needed to obtain the same effect when FU and SN-38 are combined. The CIs were calculated under the assumption of a mutually exclusive drug interaction. The combination was considered as positive (synergistic) when the combination index was <1 and negative (antagonistic) when it was >1.

### Patients

Patients with locally advanced or metastatic CRC, histologically or cytologically proven, were eligible for this study. Minimum age for enrolment was 18 years. Other requisites of eligibility were disease measurability according to RECIST criteria (Therasse *et al*, 2000); PS (ECOG) 0–2; adequate organ function: white blood cell count >3000/*μ*l, platelet count >100 000/*μ*l, Hgb>12 g/dl, creatinine <1.5 mg/dl, total bilirubin <2 mg/dl and transaminase levels <3 times upper normal limits. FU- and folinic acid (LFA)-based adjuvant chemotherapy and prior surgery for primary tumour were allowed. All patients were informed of the investigational nature of the study and expressed written informed consent. The study was approved by the local Ethics Committee.

### Clinical study design

To translate the results of the preclinical part of this study, an open-label, dose-escalating phase I trial was designed in which groups of four to six patients were to receive increasing doses of IRI and c.i. FU until dose-limiting toxicity (DLT) was demonstrated in at least two of six patients. Four dose levels of IRI and two of FU were evaluated in this study. We obtained five cohorts of patients: (i) IRI 150 mg/m^2^ and FU 800 mg/m^2^ as a 4-day c.i., (ii) IRI 200 mg/m^2^ and FU 800 mg/m^2^ as a 4-day c.i., (iii) IRI 250 mg/m^2^ and FU 800 mg/m^2^ as a 4-day c.i., (iv) IRI 250 mg/m^2^ and FU 1000 mg/m^2^ as a 5-day c.i. and (v) IRI 300 mg/m^2^ and FU 1000 mg/m^2^ as a 5-day c.i. IRI was administered intravenously as 1 h infusion, while FU was given starting 24 h after IRI administration by means of portable infusion pumps in an outpatient setting. Cycles were repeated every 3 weeks until the maximum number of 12 was reached.

Toxicity was evaluated according to NCI-CTC version 2. DLT was defined as the occurrence of grade III nonhaematological toxicity, except alopecia, asthaenia, nausea and vomiting, or grade IV neutropoenia complicated by fever or lasting for more than 5 days, or grade IV thrombocytopoenia, or a delay of more than 2 weeks in treatment due to toxicity. A minimum of four patients was observed for at least one complete cycle of combination therapy before escalating to the next dose level. In the event that fewer than two patients experienced DLT at the same dose level, a minimum of four patients were entered at the next higher dose level. All patients in the prior cohort were required to have completed one cycle of therapy before enrolment in the next cohort began. If two instances of DLT were observed at any dose level, the maximum tolerated dose (MTD) was considered to have been exceeded and a total of six patients were to be treated at the previous dose level to confirm its tolerability.

Response was evaluated after four cycles according to RECIST criteria and confirmed within 6 weeks. Patients received up to 12 cycles of chemotherapy provided they had stable disease, partial or complete response. Treatment was interrupted in the event of unacceptable toxicity, disease progression, patient refusal or physician's decision.

## RESULTS

### The synergism between SN-38 and FU *in vitro* depends on the extent of cell exposure to FU and the interval between administration of the two drugs

In preliminary experiments, we confirmed in human colon carcinoma HT-29 cells the well known evidence that the synergism between FU and SN-38 is schedule-dependent, with maximal supra-additive effect when SN-38 is administered first (Guichard *et al*, 1997; Guichard *et al*, 1998; Mullany *et al*, 1998). Cells were therefore exposed to (i) SN-38 alone for 6 h, FU alone for 24 h, (ii) the simultaneous combination of the two drugs (SN-38 and FU for 6 h followed by FU for 18 h) or (iii) both sequential combinations of the two drugs (SN-38 for 6 h and FU for 24 h and vice versa). The CI isobologram equation was used for the analysis of the interaction between SN-38 and FU (Chou and Talalay, 1984; Chou *et al*, 1994).

**Figure 1:**
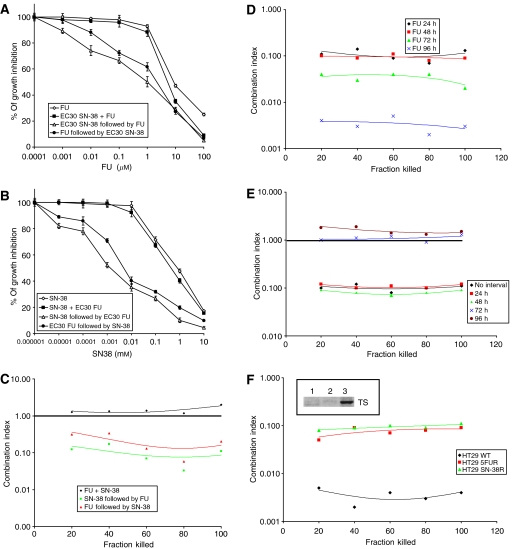
The schedule-dependent synergism between SN-38 and FU. (**A**) Cytotoxicity evaluated by MTT assay after exposure of human colon carcinoma HT-29 cells to increasing concentrations of FU (10^−4^−100 *μ*M) for 24 h, the sequential combination of EC_30_ SN-38 (0.11 *μ*M) for 6 h and increasing concentrations of FU for 24 h using both sequences and the concomitant treatment with EC_30_ SN-38 for 6 h and increasing concentrations of FU for 24 h. (**B**) Cytotoxicity evaluated by MTT assay after exposure of HT-29 to increasing concentrations of SN-38 (10^−6^−10 *μ*M) for 6 h, the sequential combination of EC_30_ FU (5.2 *μ*M) for 24 h and increasing concentrations of SN-38 for 6 h using both sequences and the concomitant treatment with EC_30_ FU for 24 h and increasing concentrations of SN-38 for 6 h. (**C**) Plot of the CIs *vs* the cytotoxicity, calculated from data reported in (**A**,**B**), using the methods described by Chou *et al* (20–21) and under the assumption of a mutually exclusive drug interaction. CI>1, antagonism; CI=1, additive effect; CI<1, synergism. (**D**) Plot of the CIs *vs* the cytotoxicity calculated from MTT assay data of HT-29 cells exposed sequentially to SN-38 for 6 h followed by FU for 24, 48, 72 and 96 h. (**E**) Plot of the CIs *vs* the cytotoxicity calculated from MTT assay data of HT-29 cells exposed to SN-38 for 6 h followed by FU for 24 h after an interval between the two drugs of 0, 24, 48, 72 and 96 h during which cells were incubated in a drug-free medium. (**F**) Plot of the CIs *vs* the cytotoxicity obtained from MTT assay data of HT-29, HT-29 SN-38R and HT-29 FUR cells exposed to the sequence of SN-38 followed by FU for 96 h. Insert: Thymidylate synthase (TS) protein expression in wild-type HT-29 (line 1), HT-29 SN-38R (line 2) and HT-29 FUR (line 3) cells.

Figure 1A illustrates the results obtained after exposure of HT-29 cells (i) to increasing concentrations of FU alone (10^−4^−100 *μ*M) or (ii) to the combination of EC_30_ SN-38 (0.11 *μ*M) and increasing concentrations of FU using both sequences or after concomitant exposure to EC_30_ SN-38 and increasing concentrations of FU. Figure 1B shows HT-29 cells grown in the presence of increasing concentrations of SN-38 alone (10^−6^−10 *μ*M), EC_30_ FU (5.2 *μ*M) and increasing concentrations of SN-38 in both sequences or concomitantly with EC_30_ FU and increasing concentrations of SN-38. As reported in Figure 1C, the maximal synergism between FU and SN-38 was achieved when SN-38 was administered before FU (green line). However, a synergism of action was also observed with the reverse sequence (red line), whereas only an additive effect was found with the concomitant exposure of HT-29 cells to both drugs (black line).

As in clinical experience IRI is always administered as a short infusion, while FU is administered either by bolus or as c.i. (Venook, 2005; O'Neil and Goldberg, 2005), we designed specific experiments to evaluate the possibility of improving the synergism between SN-38 and FU by prolonging the extent of cell exposure to FU. We therefore evaluated the synergism between a brief exposure (6 h) to SN-38 followed by a longer exposure to FU (24–96 h). As reported in Figure 1D, the synergism between the two drugs was increased by prolonging the exposure of cells to FU, reaching the maximal activity when cells were exposed to FU for 96 h (blue line).

In a third set of experiments (Figure 1E), HT-29 cells were exposed to the sequence of SN-38 for 6 h followed by FU for 24 h, but the second drug was added to the cell culture immediately after (black line) or after an interval of 24 (red line), 48 (green line), 72 (blue line) or 96 h (brown line) during which cells were incubated in a drug-free medium. Interestingly, the synergism between SN-38 and FU was unchanged whether the drug-free interval between SN-38 and FU was 24 or 48 h. In contrast, when cells were exposed to the sequence of SN-38 and FU with a drug-free interval of 72 or 96 h in between, the combination of the two cytotoxic agents obtained only additive effects.

### The sequential combination of SN-38 and FU results in an increase in apoptosis and S-phase of the cell cycle

HT-29 cells were treated with the two drugs alone or with the combination of SN-38 and FU and evaluated for the rate of apoptosis and necrosis and the cell cycle distribution. Cells treated with increasing concentrations of SN-38 (0.001–1 *μ*M) and FU (0.1–100 *μ*M) revealed a dose-dependent increase in apoptosis which is maximal at 100 nM SN-38 and 10 *μ*M FU (data not shown). Cells were exposed to 10 nM SN-38 or 1 *μ*M FU for 24 h, the combination of both drugs for 24 h or sequentially exposed to both drugs for 24 h. The sequential exposure of cells to SN-38 before FU elicited the maximal increase in apoptosis, while the reverse sequence produced intermediate levels of apoptosis compared with the simultaneous exposure (Table 1). Interestingly, when the cells were sequentially exposed to the two agents with an incubation of 96 h in a drug-free medium after SN-38, the rate of apoptosis was significantly lower and was comparable to that observed with the simultaneous exposure (Table 1).

The cell cycle distribution in HT-29 cells exposed to (i) 1 *μ*M FU for 24 h, (ii) 10 nM SN-38 for 24 h, (iii) the combination of both drugs for 24 h and (iv) the sequence of both drugs for 24 h was further evaluated. FU produced an arrest of cells in S-phase of the cell cycle, whereas SN-38 produced an arrest in the G2-M phase (Table 2). Interestingly, HT-29 colon carcinoma cells sequentially exposed to SN-38 followed by FU exhibited a significantly higher increase in the S-phase fraction with no arrest in the G2–M phase, while HT-29 cells exposed sequentially to FU followed by SN-38 exhibited an arrest of the cell cycle in both S- and G2–M phases with a magnitude similar to that produced by the single agents. In contrast, cells simultaneously exposed to SN-38 and FU exhibited an arrest of the cell cycle in the S-phase similar to that induced by FU alone, but not in the G2–M phase (Table 2).

### The synergism between SN-38 and FU is partially conserved in colon carcinoma HT-29 cells resistant to FU or SN-38

In order to evaluate whether the synergism between SN-38 and FU is able to overcome resistance to the individual agents, we obtained HT-29 cells resistant to SN-38 (HT-29 SN-38R) or FU (HT-29 FUR) and evaluated the interaction between the two drugs in both cell lines. HT-29 SN-38R and HT-29 FUR cells required concentrations of SN-38 or FU about 10 times higher than wild-type HT-29 cells to exhibit similar rates of cytotoxicity or apoptosis (data not shown). Moreover, HT-29 FUR cells exhibited increased protein levels of TS (Figure1D, inset), the molecular target of FU (van Triest *et al*, 1999), which is in agreement with the well known observation that the upregulation of TS is responsible for resistance to FU (Wong *et al*, 2001). The cytotoxicity of the sequence of SN-38 followed by FU for 96 h was evaluated in HT-29, HT-29 SN-38R and HT-29 FUR cells. In single agent-resistant cells the synergism between SN-38 and FU was still observed, albeit with a magnitude lower than that obtained in wild-type HT29 cells (Figure 1F).

### Patient population, toxicity and clinical activity

Between January 2003 and December 2004 25 patients were enrolled in the clinical study. Patients' characteristics are listed in Table 3, while toxicities are reported in Table 4. A total of 203 cycles was administered without observing treatment-related deaths. We reported three DLTs, all of which were grade III diarrhoea, in separate patients. The first patient at step 3 resumed treatment at a dose of 75% after recovering from grade II nausea/vomiting, while grade III diarrhoea occurred during the first cycle and was not repeated after dose reduction. The second and the third patients experienced grade III diarrhoea at the second and fourth cycle, respectively, and resumed treatment at full doses with no further grade III toxicities. As we did not observe a minimum of two DLTs at the same dose level, the MTD was not reached. Grade III neutropoenia was recorded in four patients and was managed with G-CSF administration. Mucositis and gastrointestinal toxicity (i.e. diarrhoea and nausea/vomiting) were the most relevant and frequently observed grade I/II toxicities; they were generally mild and rapidly reversible. The treatment was never interrupted due to toxicity.

Efficacy data are summarised in Table 5. The most remarkable result is 1 complete response, which lasted over 10 months. Twelve patients achieved a partial response (48%). Stable disease was observed in seven patients (28%), while progressive disease was recorded in five patients (20%). Overall response and disease control rates were 52% (±20, 95% CI) and 80% (±16, 95% CI), respectively. Median time to progression was 7 months (range 2–11 months).

Three patients who had received adjuvant chemotherapy were enrolled in this trial. The first had relapsed 2 months after the end of adjuvant treatment, while the other two relapsed after 24 and 48 months, respectively. The first two patients were enrolled at step 3 and the third at step 5 and, interestingly, all of them achieved a partial response. The first two patients recorded a time to further progression of 9 months, while the third, who also received radiation therapy on a iuxta-vertebral lymph-node metastasis, was progression-free at the time of this statistical analysis (11 months after the end of chemotherapy).

Most patients had a good PS at the end of treatment and all of them received second-line treatment.

## DISCUSSION

Over the past 10 years the treatment of advanced CRC has progressed dramatically, with a shift from monotherapy to combination therapy and, more recently, to sequential combination therapy (Venook, 2005; O'Neil and Goldberg, 2005; Kelly and Goldberg, 2005). The introduction of IRI and l-OHP in the first- and second-line setting has increased the complexity of delivery of care to patients. Moreover, the recent development of molecular-targeted agents that are tumour-specific and have different toxicity profiles from chemotherapeutic agents has further widened the range of therapies for this disease (Venook, 2005; O'Neil and Goldberg, 2005). As these more efficacious agents allow patients to survive longer and to receive more lines of therapy, issues have arisen concerning the choice of the best schedule and the best sequence of treatments. However, while most of the regimens combining l-OHP and FU differ only marginally, the combinations of IRI and FU are characterised by major differences in terms of doses and schedules (Venook, 2005; O'Neil and Goldberg, 2005; Tournigand *et al*, 2004; Saltz *et al*, 2000; Douillard *et al*, 2000).

In the present study we investigated, at preclinical level, the interaction between FU and SN-38, the active metabolite of IRI, in order to obtain *in vitro* evidence for optimising chemotherapeutic schedules. We observed that (i) the sequential exposure of colon carcinoma cells to the two agents produces a supra-additive effect with maximal cytotoxic activity when cells are pre-exposed to SN-38 before FU, (ii) this synergism of action is partially conserved in colon carcinoma cells resistant to SN-38 or FU, and, interestingly, (iii) it is possible to strengthen this synergism of action further by prolonging the exposure of tumour cells to FU and by administering the two agents sequentially with minimal interval in between.

Other preclinical studies previously suggested that preincubation of colon carcinoma cells with IRI before FU enhances the incorporation of FU derivatives into the DNA and DNA–protein complexes with a parallel and more persistent decrease in TS activity (Guichard *et al*, 1998). Furthermore, increased DNA damage was also observed in SW620 and HT-29 colon carcinoma cells when cells were pre-exposed to IRI before FU (Mans *et al*, 1999). These results are in agreement with our findings that the sequential exposure of colon carcinoma cells to SN-38 before FU produces a significant increase either in apoptosis or in the S-phase arrest. Indeed, while FU produced an arrest of cells in S-phase of the cell cycle and SN-38 produced an arrest in the phase G2–M, as previously reported (Mullany *et al*, 1998; Yoshikawa *et al*, 2001; McDonald and Brown, 1998), tumour cells sequentially exposed to SN-38 followed by FU exhibited a significantly higher increase in the S-phase fraction with no arrest in the G2–M phase. Thus, it is likely that preincubation of colon carcinoma cells with SN-38 facilitates in turn a more prolonged inhibition of TS by FU, an increase in the incorporation of FU derivatives into DNA, an enhanced and persistent S-phase arrest and apoptotic cell death. This hypothetical mechanism of action provides a molecular rationale to our results showing that the synergistic activity of the SN-38 and FU sequence is partially conserved in colon carcinoma cells resistant to FU and characterised by increased levels of TS. It is also in agreement with the clinical observation that the FU- and IRI-based combination therapy is effective in patients pretreated with FU (Andre *et al*, 1999) and whose tumours are generally characterised by increased levels of TS (Wong *et al*, 2001) as well as with our results obtained in three patients previously treated with FU-based adjuvant chemotherapy who achieved partial response with this modified FU/IRI regimen. Furthermore, our results suggest that IRI-resistant CRC cells may be more sensitive to schedules with c.i. FU, although the molecular mechanism of this synergism is still unclear.

The evidence that pre-incubation of HT-29 colon carcinoma cells with FU before SN-38 achieves synergism of action is partially in contrast with results reported by other authors, which suggest that the sequential exposure of cells to FU before IRI produces only additive activity (Mans *et al*, 1999). These differences may depend on the specific colon tumour cell model used.

Based on these preclinical findings and considering the low toxicity profile of infusional FU (Poplin *et al*, 2005), we designed a modified IRI/FU schedule with IRI administered on day 1 followed by a 4- or 5-day infusion of FU. We tested this alternative FU/IRI-based regimen in a phase I trial, evaluating three dose levels of IRI and two of FU in a 3-weekly schedule. Compared with the commonly used two-drug regimens (Venook, 2005), our schedule proved feasible and did not increase either haematological toxicity or the rate of high-grade diarrhoea and stomatitis. This is even more relevant in view of the toxicity profile of some traditional combination regimens of IRI and FU (i.e. IFL) in which a large proportion of patients experienced grade III–IV haematological and nonhaematological toxicity (Saltz *et al*, 2000). Although the MTD was not reached it is unlikely that this depends on the dose levels of IRI and FU. Indeed, in our modified IRI/FU schedule the theoretical weekly dose intensities of IRI and FU at the highest dose levels are 90 and 1660 mg/m^2^, respectively, very similar to the dose intensities of IRI and FU in the FOLFIRI and IFL regimens (Saltz *et al*, 2000; Douillard *et al*, 2000). Thus, taking into account that the rate of treatment delays or dose reductions reported in our study is low, it is reasonable to speculate that, at least at the highest dose levels, IRI and FU are not under-dosed.

Our regimen, tested as first-line treatment in 25 patients with advanced CRC, obtained a response rate >50%, a disease control rate of 80% and a time to progression of 7 months. These results are promising even though they were achieved in a dose-escalating phase I trial whose major aim was not to evaluate the antitumour activity. Taking into account that at the first dose level, we did not observe any response, probably because IRI was under-dosed, these findings are even more significant. Indeed, if only patients enrolled between the second and fifth dose levels are considered, the overall response and disease control rates reached 61.9 and 85.7%, respectively. Similar results were recently achieved in a phase I dose-escalating trial of IRI and c.i. FU as first-line treatment of metastatic colorectal cancer. Interestingly, the combination was well tolerated and demonstrated a significant clinical activity, obtaining an overall response rate of 55%, a clinical response benefit of 82% and a time to progression of 8 months (Saunders *et al*, 2004). Thus, the results of our study clearly suggest that the schedule of administration of the two drugs is critical to achieve the maximal supra-additive cytotoxic activity and that the lack of full synergism in some traditional schedules of IRI and FU may depend on the use of bolus FU (i.e. IFL) (Saltz *et al*, 2000) and/or the need to optimise the sequence of administration of the two agents (i.e. FOLFIRI) (Douillard *et al*, 2000).

Several options have been proposed to improve the efficacy of standard two-drug regimens for advanced CRC (Venook, 2005). Some studies have evaluated the combination of IRI, l-OHP and FU concurrently in a single regimen, the rationale being that nonspecific resistance to therapy may develop after first-line therapy. Indeed, these studies demonstrated that three-drug regimens achieve very high response rates (50–70%), but also DLTs such as neutropoenia and diarrhoea (Souglakos *et al*, 2002; Goetz *et al*, 2003). Other studies have evaluated the combination of FU with l-OHP and/or IRI administered as chronomodulated infusion. These trials also reported interesting response rates and optimal toxicity profiles, but raised questions about the feasibility of chronomodulated chemotherapy (Garufi *et al*, 2003). However, the analysis of seven phase III trials in advanced CRC suggested that exposure to all three drugs, regardless of their sequence, is a key element able to extend the overall survival of patients to 18–21 months (Grothey *et al*, 2004). Such a prospect strongly reinforces the need to optimise doses and schedules of doublet chemotherapy, in order to deliver the three drugs sequentially and obtain maximal cytotoxic activity and minimal toxicity. Moreover, these efforts seem even more relevant in the light of the introduction in the clinical management of advanced CRC of new molecular-targeted agents with a cytostatic mechanism of action requiring precise timing when combined with traditional chemotherapeutic drugs to maximise their efficacy (Venook, 2005). The combination of IRI and cetuximab represents a salvage chemotherapy in IRI-resistant patients (Venook, 2005), while the inclusion of bevacizumab in IRI/FU combination has increased both response rates and overall survival (Kelly and Goldberg, 2005). Optimising the efficacy of a combination of IRI and FU could help to enhance the overall efficacy of chemotherapy in CRC as first- or second-line therapy. Thus, this modified IRI/FU regimen may represent a precious alternative schedule with very low toxicity profile, promising clinical activity and, therefore, worth being tested in a larger phase II trial.

## Figures and Tables

**Table 1:** Analysis of cell viability in human colon carcinoma HT-29 cells exposed to different combinations of SN-38 and FU

	**Control**	**10 nM SN-38**	**1 *μ*M FU**	**SN-38+FU**	**SN-38 → FU**	**FU → SN-38**	**SN-38 → 96 h → FU**
Viable cells	92.0±1.7	87.3±1.3	84.6±1.9	71.7±1.3	59.7±1.9	66.3±2.3	74.6±1.7
Apoptosis	6.8±0.8	11.9±0.7	13.1±0.5	26.5±0.9	38.9±0.5	31.9±0.7	23.9±1.5
Necrosis	1.2±0.1	0.8±0.4	2.3±0.3	1.8±1.1	1.4±0.3	1.8±0.5	1.5±0.7

**Table 2:** Cell cycle distribution of human colon carcinoma HT-29 cells exposed to different combinations of SN-38 and FU

	**G0–G1**	**S**	**G2–M**
Control	62.8±0.8	27.0±0.9	10.2±1.0
10 nM SN-38	42.8±1.1	31.5±1.4	25.7±0.9
1 *μ*M FU	45.9±1.4	44.1±1.6	10.0±0.7
10 nM SN-38+1 *μ*M FU	40.9±1.6	46.0±0.7	13.1±1.3
10 nM SN-38 → 1 *μ*M FU	37.2±1.4	60.8±1.9	2.0±0.2
1 *μ*M FU → 10 nM SN-38	34.5±1.1	45.4±2.3	20.1±1.2

**Table 3:** Baseline characteristics of the patients

**Patients**	**25**	
*Age*		
Range	35–79	
Median	60	
	
*Sex*		
Female	7	
Male	18	
	
*PS ECOG*	N	%
0	19	76
1	5	20
2	1	4
		
*Previous surgery*		
Resection/colectomy	18	
Bypass	3	
None	4	
Adjuvant CT	5	
	
*Metastatic site*	N	%
Liver	21	84.0
Lung	3	13.0
Nodes	3	13.0
Peritoneum	4	16.0
Other	2	8.7

**Table 4:** Adverse events in patients who received different dose levels of the combination of FU and IRI

	**Level 1 (27 cycles)**	**Level 2 (29 cycles)**	**Level 3 (45 cycles)**	**Level 4 (55 cycles)**	**Level 5 (47 cycles)**	**Total (203 cycles)**
Hematological						
*Anaemia*						
Grade I–II	0	0	0	1 (1.8%)	0	1 (0.5%)
Grade III–IV	0	0	0	0	0	0
						
*Neutropoenia*						
Grade I–II	1 (3.7%)	0	0	0	0	1 (0.5%)
Grade III–IV	0	0	3 (6.6%)	1 (1.8%)	0	4 (2.0%)
						
Nonhaematological						
*Nausea/Vomiting*						
Grade I–II	3 (11.1%)	4 (13.8%)	10 (22.2%)	10 (18.2%)	9 (19.1%)	36 (17.7%)
Grade III–IV	0	0	0	1 (1.8%)	0	1 (0.5%)
						
*Diarrhoea*						
Grade I–II	8 (29.6%)	6 (20.7%)	11 (24.4%)	7 (12.7%)	9 (19.1%)	41 (20.2%)
Grade III–IV	0	0	1 (2.2%)	1 (1.8%)	1 (2.1%)	3 (1.5%)
						
*Stipsis*						
Grade I–II	0	0	0	4 (7.3%)	0	4 (2.0%)
Grade III–IV	0	0	0	0	0	0
						
*Stomatitis*						
Grade I–II	1 (3.7%)	1 (3.4%)	9 (20.0%)	7 (12.7%)	2 (3.6%)	20 (9.8%)
Grade III–IV	0	0	0	0	0	0
						
*Fatigue*						
Grade I–II	4 (14.8%)	1 (3.4%)	1 (2.2%)	2 (3.6%)	2 (3.6%)	10 (4.9%)
Grade III–IV	0	0	0	0	0	0
						
*Flu-like syndrome*						
Grade I–II	1 (3.7%)	0	1 (2.2%)	1 (1.8%)	0	3 (1.5%)

**Table 5:** Overall and partial response rates in patients who received different dose levels of the combination of FU and IRI

	**CR**	**PR**	**S**	**PD**
Step 1	0	0	2	2
Step 2	0	2	1	1
Step 3	0	4	0	1
Step 4	0	3	3	0
Step 5	1	3	1	1
Total	1	12	7	5
%	4	48	28	20
Response rate (%)	52			
Disease control rate (%)	80			
